# Amnion-Derived Multipotent Progenitor Cells Increase Gain of Incisional Breaking Strength and Decrease Incidence and Severity of Acute Wound Failure

**Published:** 2007-10-05

**Authors:** Liyu Xing, Michael G. Franz, Cynthia L. Marcelo, Charlotte A. Smith, Vivienne S. Marshall, Martin C. Robson

**Affiliations:** Department of Surgery, University of Michigan, Ann Arbor, MI; Stemnion Inc., Pittsburgh, PA; Institute for Regeneration, Repair, and Rehabilitation, Bay Pines, FL

## Abstract

**Objective:** Acute wound failure is a common complication following surgical procedures and trauma. Laparotomy wound failure leads to abdominal dehiscence and incisional hernia formation. Delayed recovery of wound-breaking strength is one mechanism for laparotomy wound failure. Early fascial wounds are relatively acellular, and there is a delay in the appearance of acute wound growth factors and cytokines. The objective of this study was to accelerate and improve laparotomy wound healing using amnion-derived multipotent cells (AMPs). AMPs' nonimmunogenic phenotype and relative abundance support its role as a cell therapy. **Methods:** AMPs were injected into the load-bearing layer of rat abdominal walls prior to laparotomy, and cell viability was confirmed. Wound mechanical properties were measured over 28 days. The incidence and severity of laparotomy wound failure was measured in an incisional hernia model. **Results:** AMP cells were viable in laparotomy wounds for at least 28 days and did not migrate to other tissues. Laparotomy wound-breaking strength was increased by postoperative day 7 following AMP therapy. AMP therapy reduced the incidence of hernia formation and the size of hernia defects. Histology suggested stimulated wound fibroplasia and angiogenesis. **Conclusions:** AMP cell therapy reduces the incidence of laparotomy wound failure by accelerating the recovery of wound-breaking strength. This results in fewer incisional hernias and smaller hernia defects.

An acute wound is defined as a traumatic loss of normal structure and function to recently uninjured tissue after a noxious insult.^1^ Acute wound healing is the highly regulated process of cellular, humoral, and molecular events activated at the time of acute injury and resulting in a time-dependent but predictable and orderly pattern of tissue repair.^2^ Unlike chronic wounds, most acute wounds heal. The problem with acute wounds is that they take a long time to heal, are meanwhile disabling, and most often result in scar.^3^

Acute wound healing failure occurs when there is an abnormality in the degree or duration of the sequential components of tissue repair.^1^ Optimizing the management of acute wounds to prevent wound failure has been a major goal of surgical science over the past 100 years. Primary strategies have relied mainly on mechanical approaches to the problem of acute wound healing failure. However, purely mechanical approaches to the problem of acute wound failure ignore the important role that the biologic response of injured tissue plays in wound-healing outcomes.^1^

Most biomechanical failures occur early on the acute wound healing trajectory at a time when wound strength is essentially zero and patients are recovering and increasing the loads placed across the abdominal wall.^4^, ^5^ The goal of most acute wound healing research is to better define the combined biomechanical mechanism of acute wound healing failure at the tissue, cellular, and molecular levels. A major focus is fibroblast activation out of quiescence and cell cycle progression after surgical injury and how this correlates with the quality of early tissue repair.^1^ Whether fascial wound failure reflects a defect in fascial fibroblast recruitment and function during incisional hernia formation or whether abnormal mechanical signals after fascial wound failure subsequently results in impaired fibroblast function is not known.^1^ Another focus to improve acute wound healing and decrease acute wound failure has been the use of cytokine growth factors which has decreased the incidence of incisional hernias in experimental models.^4^, ^6^, ^7^

Stem cells and stem cell-like multipotent cells have the ability to differentiate into many different cell types important to wound healing and tissue repair.^8^–^10^ They are also known to produce cytokine growth factors that serve as mediators to the cellular processes of the wound-healing scheme perhaps in a more “physiologic” way.^9^–^11^ Amnion-derived cells display many favorable characteristics of stem cells, including the ability to differentiate into various cell types.^12^, ^13^ They also have been shown to secrete many cytokines and growth factors.^14^–^16^ Amnion-derived cells have the additional advantages of being abundantly available, not inhibited by social, ethical, or religious strictures, and have been reported to be nonimmunogenic.^17^

The purpose of the studies reported here was to use proprietary amnion-derived multipotent progenitor cells (AMPs) provided by Stemnion Inc., Pittsburgh, PA, to attempt to accelerate the gain of surgical incision breaking strength and, in so doing, decrease the incidence and severity of acute wound failure.

## MATERIALS AND METHODS

### AMP and GFP-labeled AMP (AMP-GFP)

Proprietary amnion-derived multipotent progenitor cells (AMP) and green fluorescent protein labeled AMP (AMP-GFP) were provided by Stemnion Inc., Pittsburgh, PA. Amnion cells were harvested from placentas delivered at the time of caesarean section and processed by proprietary techniques. The cells were transported frozen and thawed immediately prior to experimental application.

### Animal model

The rat models of laparotomy healing and incisional hernias were previously reported.^18^, ^19^ Sprague-Dawley rats (Harlan Inc, Indianapolis, IN) weighing 450 to 500 g were acclimated and housed under standard conditions. Animals were allowed ad libitum intake of standard rat chow and water throughout the study. All animal care and operative procedures were performed in accordance with the *US Public Health Service Guide for the Care of Laboratory Animals* (NIH Publication No. 86-23, revised 1985) and were approved by the University Committee on Use and Care of Animals of University of Michigan.

### Laparotomy wound healing model

Briefly, a 6 × 3 cm ventral full-thickness skin flap is raised through the avascular prefascial plane, and a 5-cm full-thickness laparotomy incision is made through the linea alba at the musculo-tendinous layer of the abdominal wall. The laparotomy is repaired with a running, 4-0 polypropylene suture using 0.3 cm suture bites and 0.5 cm progress between stitches. The suture is tied to itself at the end of the wound. The skin flap is sutured in place with three 4-0 polypropylene stitches and steel wound clips. After 30 minutes of recovery under a heat lamp, the rats are returned to fresh individual cages.

**Figure 1:**
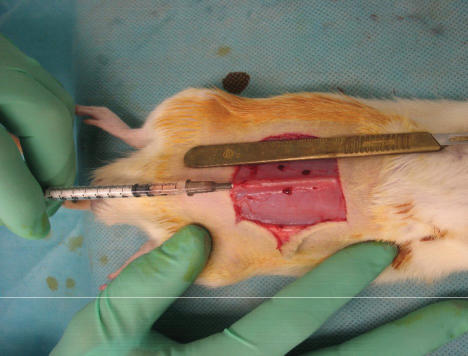
Delivery of NS or AMP suspension in NS into linea alba. 200 μ L NS or AMP NS suspension was injected along 5 cm of the linea alba using a 22G needle (0.7 mm × 38 mm). Even tissue distribution was achieved.

Two groups were studied: (1) a normal saline treated (NS-control-S) abdominal wall group and (2) a NS washed, human amnion-derived multipotent progenitor cell primed (AMP-S) abdominal wall group. In the NS control group, the midline of the abdominal wall (linea alba) was injected for 5 cm with 200 μ L of NS (priming). In the AMP-S group, 200 μ L NS containing 10^6^ amnion epithelial cells was also injected along the linea alba for 5 cm. Surgical site priming is achieved with a 22-gauge hypodermic needle (0.7 mm × 38 mm) into the linea alba (Fig. 1). Soft tissue distribution of NS control or AMP cell suspension was achieved. After 5 minutes, the laparotomy incision is made and repaired as described above, closed without laparotomy.

On postoperation day (POD) 7, POD 14 and POD 28, the rats were killed. Isolated abdominal wall muscle and tendon strips and fresh biopsies of the abdominal wall to laparotomy wound-healing interface were collected for mechanical and histological testing.

### Laparotomy wound breaking strength and mechanical properties

Mechanical testing was performed on abdominal wall strips collected from the laparotomy wound healing model as reported.^6^, ^19^, ^20^ All sutures were removed. Abdominal wall strips were taken perpendicular to the wound-healing interface. A cutting template was used to mark the abdominal wall to minimize size variability between specimens. Strips were cut 10 mm in width, 60–80 mm in length. Two strips were collected from each rat, and testing was performed within 6 hours of necropsy. The fascial tissue strip thickness at the wound and the length between grips were measured with Digimatic calipers (Mitutoyo American Corp, Chicago, IL). Stretch loading facilitated mechanical characterization of the wound-healing interface. Force extension curves were generated for each fascial strip with the use of an Instron tensiometer (model 5542; Instron Corporation, Canton, MA) equipped with a 50-newton static load cell set at a crosshead speed of 10 mm per min. The fascial strips were mounted into the load frame with the use of pneumatic grips, preloaded to 0.1 newtons with the gauge length measured between the grips that was around 10 mm. The load frame applied testing loads to the fascial strips until mechanical tissue disruption occurred. The anatomic location of the break was noted for each specimen. Force and tissue deformation data were simultaneously recorded and captured on a computer connected to the load frame via a digital interface card. Data analysis were performed with the use of the Merlin materials testing software package (Instron Corp, Canton, MA) from which breaking strength, the maximum load force (*F*_max_) at mechanical failure (N); tensile strength, the maximum stress developed in the specimen per unit area, calculated as *F*_max_/cross-sectional area (N/mm^2^); energy at break (mJ), yield strength (N), yield energy (mJ), and stiffness, the slope of the linear elastic region of the force-extension curve (N/mm) were generated with the Merlin software package. Failure of the specimen was defined at the yield point, rather than at the point of ultimate tissue disruption, because the yield point indicates the region where the tissues are irreversibly deformed (ie, beyond the elastic limit of the tissues).

### Incisional hernia model

Failing laparotomy incisions form incisional hernias. Clinically, this manifests as defects in the muscular-fascial-peritoneal layer of the abdominal wall. The most serious consequences of this are the incarceration and obstruction of abdominal viscera, loss of the ability of the abdominal wall to maintain torso posture, and chronic pain.

The models were made as previously described.^18^, ^19^ Briefly, a 6 × 3 cm ventral full-thickness skin flap was raised through the avascular prefascial plane, and a 5-cm full-thickness laparotomy incision was made through the linea alba. Two 5-0 fast absorbing plain gut stitches were placed on top end and middle point of the laparotomy incision, respectively. The skin flap is sutured in place with three 4-0 polypropylene stitches and steel wound clips. Two groups were again used for this experiment: normal saline primed (NS-H control) abdominal wall group and human amnion-derived multipotent progenitor cell primed (AMP-H) abdominal wall group. In the NS-H control group, 200 μ L NS was injected along the midline for 5 cm. In the AMP-H group, 200 μ L NS containing (1–5) × 10^6^ amnion epithelial cells was injected along the linea alba for 5 cm. On POD 28, the rats were killed and the musculo-tendinous layer of the abdominal wall was collected and examined for incisional hernias.

### Measurement of hernia size

**Figure 2:**
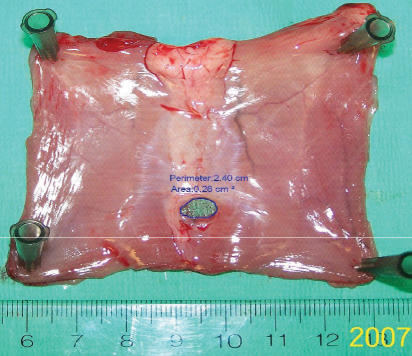
Measurement of hernia size. Digital pictures were taken with a ruler as reference for every sample. Software Spot was employed to measure the calibrated hernia sizes. A digital perimeter was drawn along the hernia ring to measure the hernia size in square centimeters.

For hernia size measurement, the hernia model rats were killed on POD 28. The skin was dissected free circumferentially and 5 cm × 10 cm of the abdominal wall muscle was excised. The muscle was stretched out and pinned down on a dissecting board at the four corners with the peritoneal side up. A ruler was set alongside the wound as a reference for every sample. A standardized digital picture was the taken. Software Spot, Windows: version 4.5 (Diagnostic Instruments, Inc., Sterling Heights, Michigan, USA) was employed to calculate the hernia size on digital pictures. Calibration was set up using the rule reference on each picture. A circle was drawn along the hernia ring to measure the hernia size as square centimeter (Fig. 2).

### Wound and tissue histology

Sagittal fascial laparotomy wound and/or hernia sections were then cut and immediately fixed in 10% neutral-buffered formalin in preption for histologic analysis. Specimens were embedded in pffin, sectioned, and stained with hematoxylin and eosin or trichrome by a core research histology service or the Immunoperoxidase Laboratory in the University of Michigan Comprehensive Cancer Center.

Viable green fluorescence protein labeled AMP (GFP-AMP) was measured in the linea alba, spleen, liver, skin, lymph tissue, and testis. GFP-AMP were injected into the linea alba as described above and animals killed on POD 7, 14, and 28. Tissues were collected and embedded in Optimal Cutting Temperature compound (OCT, Sakura Finetek, Torrance, California) and snapped in liquid nitrogen immediately. A piece of sagittal fascial wound from the linea alba was fixed in 105 formalin on ice for 6 hours, dehydrated through graded sucrose washes for 24 hours, and finally embedded in OCT compound and snapped in liquid nitrogen. Serial cryostat sections were made for fluorescence microscopy to evaluate distribution or localization of GFP+ cells.

### Statistics

Statistic analysis was performed using GraphPad Prism version 4.00 for Windows (GraphPad software, San Diego, CA, www.graphpad.com). *t*-Test was used to compare the difference between NS control group and AMP-treated groups. This software was also used to create the incidence curves of incisional hernia at POD 28 for fractional hernia at any particular hernia or wound defect size, and compare the curves between NS-H control and AMP-H groups. Significant level was set at *P* < 0.05.

## RESULTS

### Viability of GFP-labeled AMP in experimental wound and hernia models

Successful transplantation requires survival of the transplanted AMP. Reports suggest that amniotic cells were beneficial for the healing of spinal injury and do not induce immune rejection reaction.^17^, ^21^–^23^ Human amniotic epithelial cells have not been reported to express on their surfaces HLA-A, B, C, and DR antigens, or beta 2-microglobulin.^21^ Human amnion epithelial cells have been reported to survive up to 7 weeks without inducing acute immune rejection after allogeneic transplantation of human amniotic epithelial cells. Yeager and colleagues reported that no residual amniotic epithelium was found in humans at 2 1/2 and 3 1/2 months after human amniotic cell implantation.^24^

**Figure 3:**
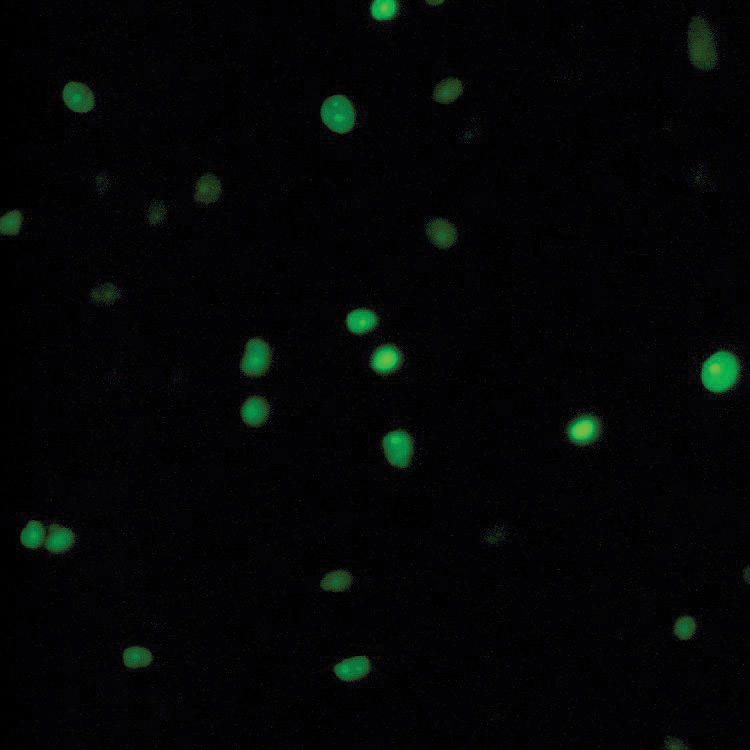
Cultured GFP labeled human AMP (AMP-GFP).

**Figure 4:**
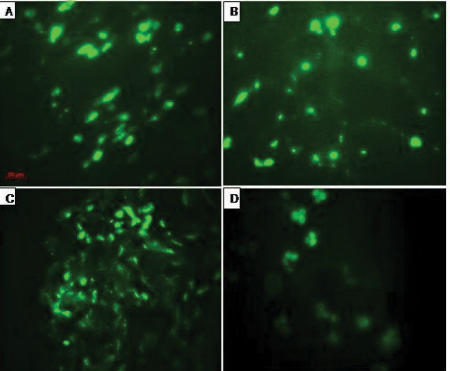
Viability of human AMP-GFP in laparotomy wounds. AMP-GFP were injected in rat linea alba and tissue harvested over 7, 14, and 28 days. Frozen tissue sections of abdominal wall collected at these three time points were examined under a fluorescent microscope. A: POD 7; B: POD 14; C: POD 28. D: Frozen skin tissue section adjacent to injection site collected on POD 7.

To evaluate the survival of AMP in our models, 2.5 × 10^6^ GFP-labeled AMP were injected into the linea alba (Fig. 3). Rats were killed on POD 7, 14, and 28. Abdominal wall tissue around the linea alba injection site was collected. Frozen tissue sections were made for fluorescence microscopy examination. As shown in Figure 4, high numbers of the cellular elements were found to express GFP in abdominal wall tissue sections of rat on POD 7, 14, and 28, suggesting AMP survive within this experimental time frame. To evaluate whether the AMP migrate outside the wound site, tissues of liver, spleen, lymph nodes, testis, and skin wound adjacent to the laparotomy delivery site were collected and checked under fluorescent microscope for GFP expression. No GFP expressing cellular elements were found in the liver, lymph nodes, spleen, and testis (data not shown). As shown in Figure 4D, GFP positive cellular elements were found in skin specimen adjacent to the AMP delivery site, suggesting the possibility that AMP could spread locally but not migrate to distant tissue.

### AMP accelerates wound healing in the early postoperative laparotomy incision

**Figure 5:**
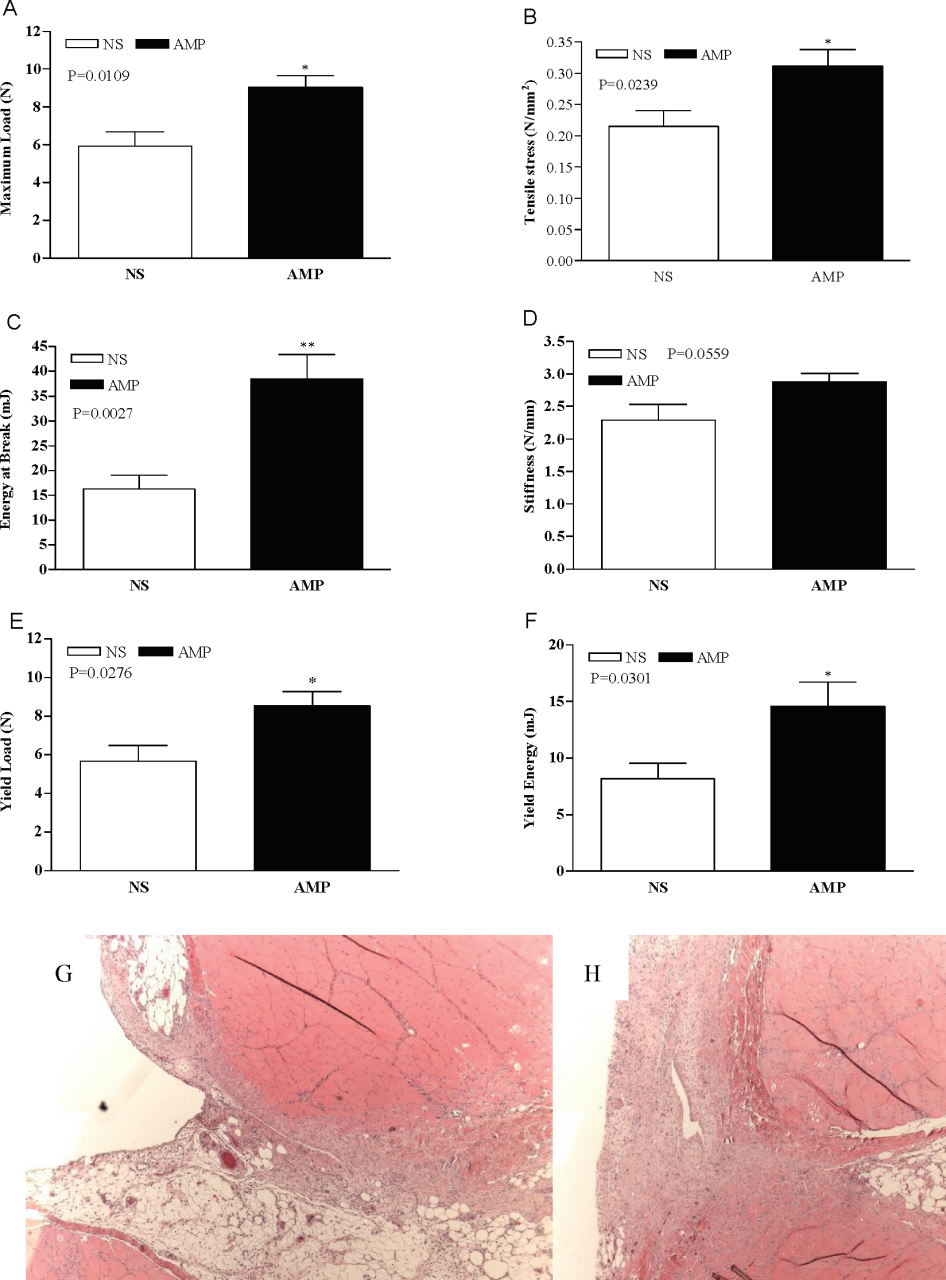
Tensiometric measurements and histology examination on early postoperative incisions. Fascial mechanical breaking characteristics were measured with an Instron tensiometer on POD 7 (A to F). Values are the mean ± SEM of 6 wound biopsies each from the NS-S control and the AMP-S groups. *t*-Test was employed to compare the difference between the 2 groups. *: *P* < 0.05, **: *P* < 0.01. Morphology of fascial incisional wound at POD 7 from NS-S (G) and AMP-S (H) groups. H&E staining was performed for histology.

According to the published literature, amniotic cells may facilitate the recovery of injury in the nervous system.^17^, ^23^, ^25^, ^26^ In this experiment, AMP were delivered to the linea alba to test AMP's effect on abdominal wall muscle-tendon wound healing. Tensiometric analysis was performed on uniform abdominal wall strips with the line of tissue deformation directly perpendicular to the linea alba/incision line. Tensiometric measurements found significant differences in the mechanical properties between the NS-S and AMP-S groups at the early phase of wound healing. All wounds mechanically disrupted at the fascial: fascial interface. As shown in Figure 5, wounds treated with 10^6^ AMP/rat developed increased breaking strength in the early postoperative fascial incision (POD 7). The scar tissue harvested on POD 7 showed evidence of higher vascularization, more granulation tissue, and organized fibro-proliferation in the AMP-S group compared with NS-S group (Figs. 5F and G).

**Figure 6:**
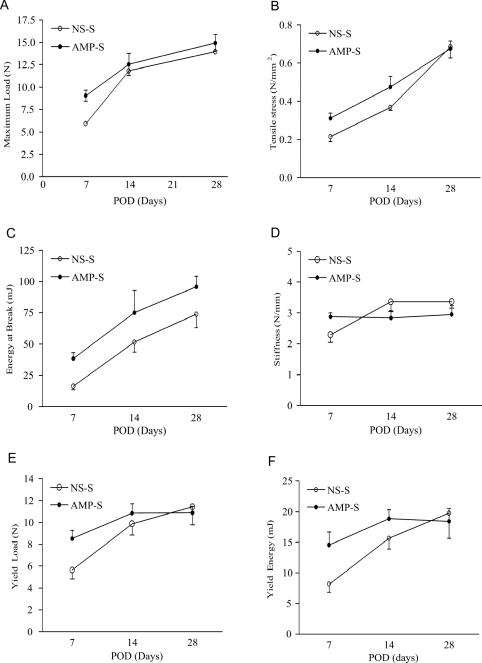
Time course of mechanical wound breaking property recovery. Fascial mechanical breaking characteristics were measured with an Instron tensiometer on 3 time points, POD 7, POD 14, and POD 28. Values are the mean ± SEM of 6 wound biopsies each from the NS-S control and the AMP-S groups.

To investigate AMPs' long-term effect, the period of the experiments was extended to POD 28. By postoperative 28 day, the mechanical wound-breaking characteristics increased along with time in both groups. Differences in the recovery of wound-breaking properties were decreasing on POD 14 and appeared to become less marked with time, consistent with normal acute wound healing (Fig. 6). There was no statistical difference in wound-breaking strength or tensile stress on POD 14 and 28 following single dosing of AMP.

### AMP reduces laparotomy dehiscence and herniation

**Figure 7:**
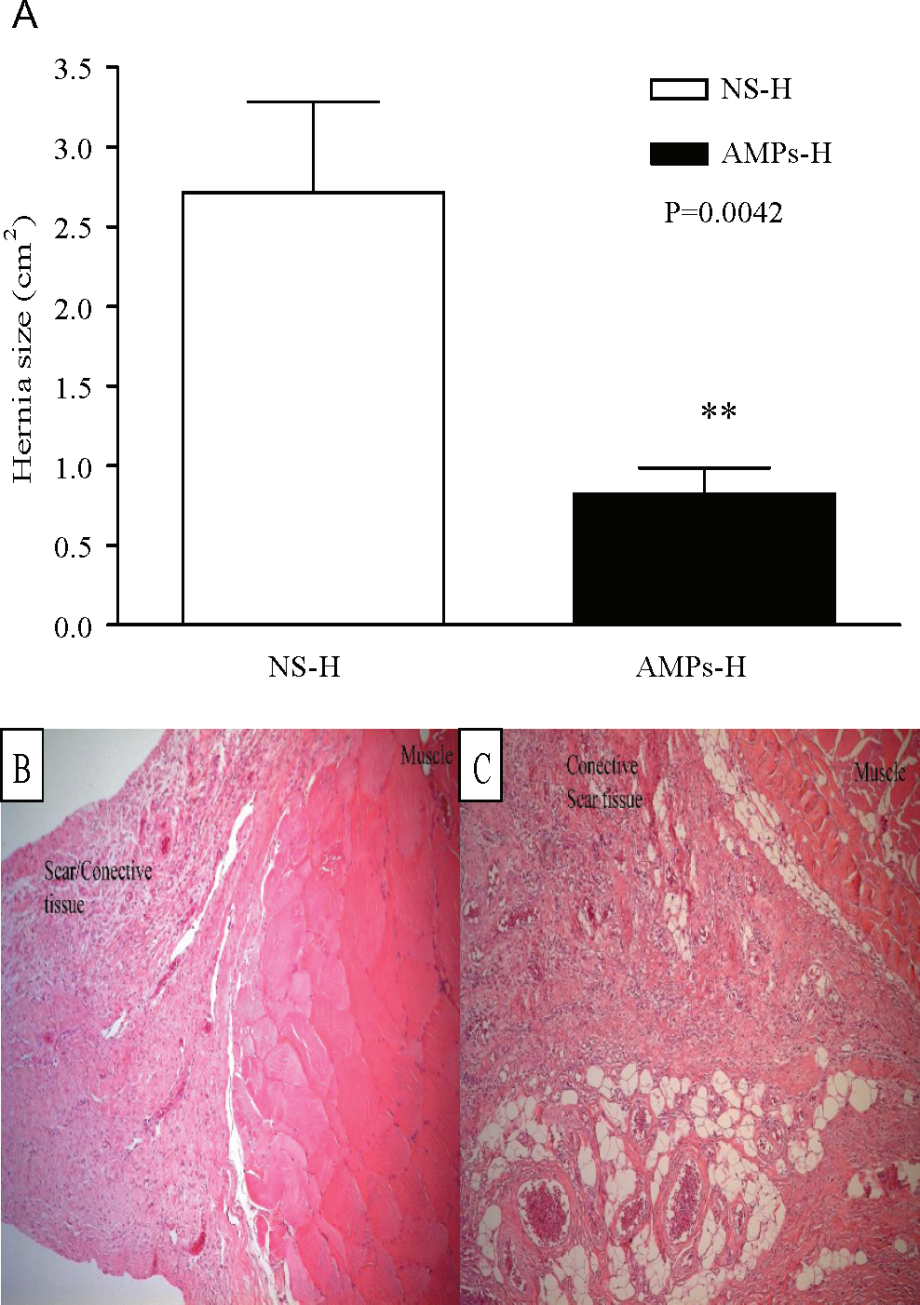
Effect of AMP on hernia healing. Hernia or wound defect size was measured on POD 28. Values are the mean ± SEM of 11 hernia biopsies each from the NS-H control and the AMP-H groups. **: compared with NS-H, *P* < 0.01. Morphology of hernia ring from NS-S (B) and AMP-S (C) groups. H&E staining was performed on tissue sections.

Initial experiments showed that AMP augmented wound healing in the early postoperative fascial incisions. Incisional hernia of the laparotomy incision is a very common complication of the human abdominal wall. To test whether AMP improved laparotomy healing and hernia formation, rat hernia models were treated with AMP cells. (1–5) × 10^6^ AMP in 200 μ L NS or 200 μ L NS were delivered on linea alba over a 5-cm length. A 5-cm fascial laparotomy incision was made and closed with two fast absorbing suture stitches resulting in an intentional incisional hernia model. On POD 28, all rats were killed and the abdominal wall was collected for hernia or wound defect assessment and size measurement. As shown in Figure 7A, the hernia or wound defect size in AMP-H group was significantly smaller than those in NS-H group, 0.82 ± 0.16 cm^2^ versus 2.72 ± 0.56 cm^2^. Morphology of hernia ring showed (Figs. 7B and C) that there was more vascularization within the hernia ring from AMP-H treated rats when compared with the NS-H rat, suggesting accelerated and improved laparotomy wound repair in the AMP-H group.

In the best clinical study of predicting incisional hernias, it was found that a 12-mm gap in the laparotomy closure on POD 30 would go on to form a clinically significant incisional hernia 94% of the time. Conversely, defects less that 12 mm in size on formed important incisional hernias 3% of the time over 3 years.^27^ In other words, small laparotomy defects do not form incisional hernias, but bigger ones do. The majority of AMP-treated animals developed very small laparotomy defects, if at all. The largest and smallest hernia or defect sizes were 1.88 and 0 cm^2^ in AMP-H group versus 6.28 and 0.50 cm^2^ in NS-H group, respectively. AMP treatment, therefore, reduces the incidence of laparotomy wound failure that progresses to an incisional hernia.

## DISCUSSION

As stated previously, acute wound failure is a biomechanical failure. When incisions of the musculo-tendinous layer of the abdominal wall fail, incisional hernias form. The most serious consequences of this are the incarceration and obstruction of abdominal viscera, loss of the ability of the abdominal wall to maintain torso posture, and chronic pain. Mechanical approaches to laparotomy closure, including optimized suture length-to-wound length ratios, incision location and orientation, layered versus mass closure, and even mesh herniorrhaphy, have all failed to eliminate this common surgical complication.^28^, ^29^

The two most likely mechanisms for acute fascial wound failure leading to incisional hernia formation have been summarized as follows: a defect or delay in repair cell activation and provisional wound matrix crystallization during acute fascial repair leads to herniation, or herniation resulting from mechanical failure leads to a delay or deficiency in the acute wound healing process.^1^ It is possible that both mechanisms are active in a reinforcing cycle of acute wound failure and herniation.

The injured tissue's mechanical integrity is best measured as wound-breaking strength. Wound-breaking strength is the mechanical property of a healing wound and measures the ability of the early scar to resist distractive forces. Burst abdomens, or acute fascial dehiscence with evisceration, are one important extreme of acute wound failure. Burst abdomens have long been associated with mortalities of almost 50%.^28^ Less dramatic are incisional hernias, another example of acute fascial wound healing failure that is a large source of surgical morbidity. The magnitude of the problem is emphasized by the nearly 50 million operations performed each year in the United States.^30^ This number does not include the annual estimated 50 million traumatic wounds.^1^ Recovery from these procedures and injuries costs 250 million patient-days in lost productivity and billions of dollars in lost or supplemental earnings. Despite technical advances, the incidence of fascial dehiscence, incisional hernia formation, gastrointestinal anastamotic breakdown, pancreatic fistulas, and vascular pseudoaneurysms has not declined substantially in 75 years of modern surgery.^28^ Prospective, well-controlled studies find that incisional hernias comprise 11% of all abdominal wall closures after celiotomy.^31^, ^32^ In as many as 1 in 3 abdominal wall closures, the fascial layer of the wound will fail to heal reliably in patients operated upon for aortic aneurysm disease during periods of hemodynamic instability or in those with gross contamination of the deep wound space, especially in malnourished patients.^28^ As a result of such problems, approximately 200,000 incisional hernia repairs are performed each year in the United States alone at a financial cost of nearly $ 2.5 billion.^30^, ^33^ The cost in terms of prolonged recovery times and patient disability is more difficult to measure.^34^ With nearly 4 million abdominal and pelvic operations performed each year in the United States, it is estimated that another 200,000 incisional hernias may be going unrecognized and untreated.^6^

There are several potential mechanisms by which AMP cells improve the healing of laparotomy incisions. AMP cells may differentiate into fibroblast-like cells to replace or augment the fibroblasts made defective by biomechanical stress when the single suture gives way. Defects have been identified in the kinetic properties of fibroblasts cultured from fascial biopsy specimens.^35^ In these studies, it was observed that fibroblasts cultured from incisional hernias were significantly deficient in causing contraction of fibroblast-populated collagen lattices. Normally healing fascial fibroblasts caused 80% lattice contraction over 5 days, whereas hernia fibroblasts caused only 50% lattice contraction.^35^ Other groups observed abnormal collagen isoform production in patients with incisional hernias. It is possible that a regenerative signal from AMP cells directs accelerated and or normal fascial fibroblast repair.

AMP cells could be making the necessary cytokine cocktail at the proper time and dose to effect a trajectory shift—already proven by our group for individual cytokines such as TGF-B2, bFGF, and GM-CSF.^2^, ^4^, ^35^ The immune privilege of amnion-derived cells allows them to function when applied as an allograft or xenograft.^17^

Tissue growth factors are an important class of tissue repair signaling peptides upregulated during the lag phase of acute wound healing.^2^ Five to seven days are required, however, before peak levels of fibroproliferative growth factors like transforming growth factor beta are reached in acute wounds.^36^ Acute wound therapy with proliferative growth factors is known to accelerate the appearance of fibroblasts and collagen into the wound, thereby shortening the natural lag phase for gain in injured tissue strength.

The preponderance of clinical and basic evidence now supports the concept that very early laparotomy wound failure is the mechanism of incisional hernia formation. Load-bearing laparotomy wounds fail at the weakest point on the acute wound healing trajectory, at the same time that most surgical patients are recovering. Early wound failure lead to incisional hernia formation more than 94% of the time.^27^ Well-performed, prospective series of incisional hernias now report early recurrences, all within 3 years of operation. It is increasingly accepted that a majority of incisional hernias arise from occult fascial dehiscences. A new emphasis is, therefore, being placed on improved laparotomy closure and healing, either through biological manipulation, or with the use of prophylactic, soft-tissue prostheses. Preclinical work has confirmed that accelerated laparotomy repair through priming with tissue repair growth factors can significantly reduce the incisional hernia rate.^4^, ^6^ The problem with single peptide wound therapy remains drug delivery, dosing, and timing.

Histological samples of AMP-treated wounds are consistent with accelerated repair over controls. There is more fibroplasia and angiogenesis. It is also suggested that the intensity of the wound inflammatory response is modulated and less severe. Collagen fibrils appear more organized along natural lines of tension and the peritoneal lining appears restored during AMP treatment.

A comprehensive examination of remote organs and tissue found no migration or trafficking of AMP cells beyond the wound. This supports the application of AMP cells in wound therapy. The immune privilege of amnion-derived cells permits its use as an allograft and the containment of these cells to the wound suggests its safety.
